# Simple strategy for preparing nanoporous silver sheets as reusable SERS substrates for trace analysis with up to 60 reuses

**DOI:** 10.1007/s00216-025-05903-2

**Published:** 2025-05-22

**Authors:** Hongni Zhu, Vince St. Dollente Mesias, Xin Dai, Wenting Qiu, Xiaobin Yao, Jinqing Huang

**Affiliations:** 1 Department of Chemistry, The Hong Kong University of Science and Technology, Clear Water Bay, Hong Kong, China; 2 Research Center for Biomedical Optics and Molecular Imaging, Key Laboratory of Biomedical Imaging Science and System, Shenzhen Institutes of Advanced Technology, Chinese Academy of Sciences, Shenzhen, 518055 China

**Keywords:** SERS, Nanoporous, Silver sheet, Reusable, Pesticides

## Abstract

Leveraging fingerprint-like specificity of molecular vibrations and enhanced sensitivity from metallic nanostructures, surface-enhanced Raman spectroscopy (SERS) has emerged as a powerful analytical tool. However, most SERS substrates are designed for disposable single-use, which compromises data reproducibility, increases operational costs, raises sustainability concerns, and narrows practical applications. Furthermore, the complex fabrication methods of existing reusable SERS substrates and their limited recycling/reusable cycles pose significant challenges to address the needs of automated high-throughput analytical applications. In this study, we developed a novel nanoporous silver sheet as a background-free, highly sensitive, and reusable SERS platform, using a simple chemical redox method. With multiple layers of uniform coral rock–like nano-silver rods, it demonstrated excellent sensitivity with a detection limit of 1 × 10⁻⁷ M (24 ppb) for thiram and universal detection capability across a diverse range of 10 pesticides, such as carbamates, organophosphorus compounds, pyrethroids, organochlorines, and benzimidazoles, which was further validated through practical testing on yellow cabbages. More importantly, this nanoporous silver sheet could be reused at least 60 times from a straightforward rinsing procedure to maintain its consistently outstanding performance for continues SERS measurements. Given its accessibility and economic viability, this highly reusable SERS substrate holds the promise to promote the practical applications of SERS in various fields in the future.

**Graphical Abstract:**

A simple chemical redox method enabled a robust SERS detection platform with high sensitivity, reproducibility, and reusability for 60 cycles.

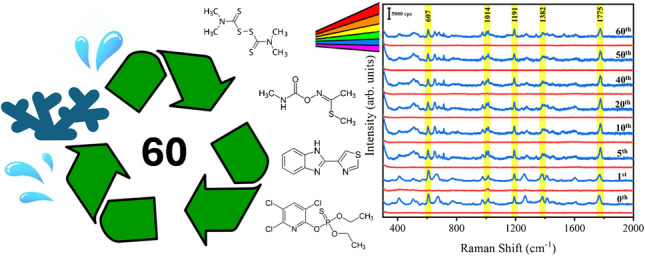

**Supplementary Information:**

The online version contains supplementary material available at 10.1007/s00216-025-05903-2.

## Introduction

Surface-enhanced Raman spectroscopy (SERS) is a versatile analytical technique that offers remarkable specificity and sensitivity, demonstrating significance across various fields, such as food science, environmental monitoring, material characterization, biomedical diagnostics, and forensics [1]. It leverages distinctive molecular vibrations from Raman spectroscopy to enable specific chemical identification, while overcoming sensitivity limitations for molecules with low inelastic scattering cross-sections [2]. Utilizing the localized surface plasmonic oscillations of noble metal nanostructures, the local electromagnetic field can be boosted up with ~ 10 orders of magnitude in SERS measurements, which makes single-molecule detection possible [3, 4]. However, current SERS substrates are primarily designed for disposable usages, which compromises data reproducibility, increases operational costs, raises sustainability concerns, and limits its practical applications. While a few reusable and durable SERS substrates have been reported, their performances require further development and investigation to maximize the potential of this powerful technique.

The most accessible and commonly used SERS substrate fabrication method involves chemically reduced metal colloids [5, 6]. These colloids, typically made of silver or gold, are deposited onto a surface, such as a plate or the object being analyzed, to form a thin film as the SERS detection platform, which may be easily washed away by liquids used in experiments. An alternative approach involves growing metal nanoparticles directly on the surface of target objects to enhance adhesion. For instance, Xu et al. fabricated ZnO@Au nanorods on FTO glass for detecting organic pollutants like methyl blue and crystal violet [7]. Au nanoparticles were reduced and grown on ZnO nanorods, forming dendritic nanostructures that can be reused up to 20 times, though the signal intensity decreased by 35% and the substrate was regenerated by triggering photocatalytic reactions on the surface of ZnO using a 365-nm laser to avoid liquid rinsing. Another approach employs physical deposition methods, such as physical vapor deposition or sputtering to deposit nanoparticles onto substrates [8, 9]. For example, He et al. developed a graphene-Au–Ag-porous GaN substrate that was functionalized with Cy3-DNAzyme to capture Pb^2+^. Au and Ag nanoparticles were deposited on the porous GaN surface using a thermal evaporation technique, which exhibited a detection limit down to 1 nM Pb^2+^ and could be reused up to three times without significant loss in signal intensity [10]. Besides, chemical approaches enable stronger interactions between nanoparticles and substrates. For example, Guo et al. developed a 4-pyridinethiol/Au nanoparticle/ITO chip for detecting Hg^2+^ ions, where Au nanoparticles are chemically immobilized using inositol hexaphosphate [11]. This substrate was reported to be reusable up to five times after rinsing with EDTA solution. Notably, single-layer film-based substrates have limited enrichment capabilities and detection sensitivity. Consequently, there is a growing trend toward fabricating more complex 3D heterostructures with higher specific surface areas to enhance analyte capture and signal amplification. However, the fabrication methods for these reusable SERS substrates are sophisticated, posing significant challenges in accessibility, reproducibility, scalability, and cost-effectiveness [12]. More importantly, the number of reliable recycling/reuse cycles for these substrates is typically not high enough, which is a critical factor for automated high-throughput analytical applications.

In our previous work, a nanoporous silver sheet was fabricated using an electrochemical method, which could be reused for more than 20 cycles [13]. Nevertheless, a few noisy signals in the background arising from side reactions in the electrochemical fabrication process hinders its performance. The universal detection capability across a diverse range of analytes remains limited, which is a crucial requirement for on-site, large-scale, multi-pesticide detection in real-world applications. In this study, we developed a new nanoporous silver sheet using a simple chemical redox method, which could serve as a background-free, highly sensitive, and reusable SERS platform. With multiple layers of uniform coral rock–like nano-silver rods, it demonstrated excellent performance in detecting various pesticides, achieving a detection limit of 1 × 10⁻⁷ M (24 ppb) for thiram, as validated through practical testing on yellow cabbages. More importantly, it could be reused at least 60 times through a straightforward regeneration procedure, maintaining robust performance for continues SERS measurements. Considering the facile fabrication, excellent sensitivity, and high reusability, this nanoporous silver sheet holds great potential to enhance the adoption and practical application of SERS-based analytical techniques.

## Materials and methods

### Chemical reagents

All chemicals were used without further purification. Silver foil (Ag, 99.9% and 0.127 mm thick) was purchased from Alfa Aesar (MA, USA). Thiram, phosmet, carbaryl, methomyl, deltamethrin, chlorpyrifos, fenvalerate, dieldrin, thiabendazole, acephate, and sodium borohydride were purchased from Sigma-Aldrich (St. Louis, MO, USA). Ethanol, 30% hydrogen peroxide, and 37% hydrochloric acid were purchased from BDH Chemicals Ltd. Milli-Q water (resistivity of 18.2 MΩ cm) was used for sample preparation and SERS measurement.

### Preparation of nanoporous silver sheet

Ten milliliters of hydrochloric acid (37%) was first mixed with 500 µL of hydrogen peroxide (30%). Then, the pure silver foil (99.9%) with a size of ~ 1 cm × 2 cm was washed with distilled water and put into the mixture for ~ 18 h. The color of the mixture became yellow, and the metal surface turned from lustrous silver into grey. After that, the liquid was discarded, and the sheet was washed with distilled water for three times and quickly placed into the 0.1 M sodium borohydride solution. The metal surface turned from grey into white. The nanoporous silver sheet was obtained after visible bubbles had stopped appearing in the solution. Finally, the sheet was washed with water three times and preserved in the sealed bottle with inert gas. The nanoporous sheet has a thickness of ~ 0.11 mm. In the following SERS experiments, the sheet was further cut into small pieces with a size of ~ 5 mm × 5 mm.

### Characterization of nanoporous silver sheet

The morphology of the nanoporous silver sheet was measured using scanning electron microscopy (SEM, JSM-6390, JEOL, USA Inc.). The sizes of nanopores were counted by ImageJ. The background spectra were acquired by a confocal Raman microscope (Renishaw, Gloucestershire, UK) with a 633-nm laser and a 20 × microscope objective. The laser power was 5 mW and the acquisition time was 30 s. All spectra were analyzed after baseline correction by Renishaw WIRE (v4.0, UK).

### Preparation of pesticide solution

The pesticides (thiram, phosmet, carbaryl, methomyl, deltamethrin, chlorpyrifos, fenvalerate, dieldrin, thiabendazole, acephate) were dissolved in ethanol to prepare the stock solution (10 mM), respectively. Solutions with different concentrations for SERS measurements were prepared by appropriate dilution of each stock solution with water.

### SERS measurements

To check the detection sensitivity (Fig. 2) and generality (Fig. 3), the nanoporous silver sheet was immersed in the pesticide solutions for ~ 18 h to ensure the complete adsorption of pesticide molecules onto the substrate surface. The sheet was then removed from the solution, dried at room temperature, and subjected to perform SERS measurements. For reusability trials, the soaking time of phosmet solution was reduced to 5 min, then the nanoporous silver sheet was totally immersed in ethanol for 4 h with continuous stirring to remove the attached pesticide. The cleaned nanoporous silver sheet was dried in a fume hood for the next detection. The cleaning steps were repeated after each SERS measurement. All SERS spectra were acquired with the laser power of 5 mW and the acquisition time of 30 s from a confocal Raman microscope (Renishaw, Gloucestershire, UK) with a 633-nm laser and a 20 × microscope objective. All spectra were analyzed after baseline correction by Renishaw WIRE (v4.0, UK).

### Detection of pesticides on vegetables

The test yellow cabbage was purchased from a local market and washed with double distilled water before use. Then, the peels were cut into uniform-sized squares 1 × 1 cm^2^. After that, 100 μL of thiram solution (1 × 10^−6^ mol L^−1^) was dropped onto the surface of the peels and dried at room temperature. To extract and concentrate pesticide residues, 20 μL of ethanol was dispersed onto each above-treated sample peel. After 2 min, the solution on the peels was collected into a small tube and the nanoporous silver sheet was totally immersed in the solution for ~ 18 h to ensure complete adsorption and optimal detection of thiram. The nanoporous silver sheet was then placed on a glass slide and dried at room temperature for subsequent SERS analysis.

## Results and discussion

**Fig. 1:**
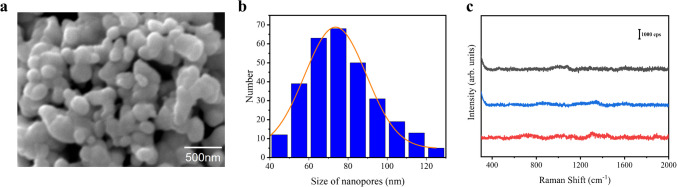
**a** SEM image of a nanoporous silver sheet. **b** Size analysis of nanopores. **c** Background spectra of the nanoporous silver sheet at different locations

As shown in Fig. 1a, the surface texture of a nanoporous silver sheet shows a coral-like structure with abundant nanopores. Figure 1b shows the size distribution of these nanopores as determined by SEM images collected from three different locations (Figs. 1a and S1), averages between 70 and 80 nm in width. These nanopores greatly extended the effective depth under the surface of the nanoporous silver sheet, providing numerous active SERS hotspots to enlarge the volume of analyte and reduce the detection concentration, thereby improving sensitivity for SERS measurement. Since the uniformity of SERS signals is highly related to structural stability, spatial scanning across different locations of the nanoporous silver sheet for generating the background spectra can be used to verify the stability of the SERS substrate. As illustrated in Fig. 1c, the background spectra of the nanoporous silver sheet at different locations are identical in peak positions and intensities, indicating the stability of this SERS substrate. Moreover, the background noise was very weak without distinguishable peaks, which was assumed to the clean reduction and cleaning procedures to avoid the introduction of impurities. In our following experiments, it was confirmed that this weak background noise caused little interference to the SERS signals of analytes and contributed to the high sensitivity in detecting trace amounts of analytes.

Nowadays, SERS has been introduced to be a powerful tool in detecting trace amounts of analytes, such as pesticide residues [14]. Trace amounts of pesticides from excessive use can be accumulated throughout the food chain, which probably poses a threat to human health [15]. Until now, various techniques have been applied in the detection of pesticides, such as liquid chromatography coupled with mass spectrometry (LC–MS) [16], gas chromatography coupled with mass spectrometry (GC–MS) [17, 18], high-performance liquid chromatography (HPLC) [19], and immunoassay [20]. These conventional methods have been considered as the “gold-standard” of analytical methods for pesticide detection due to their high sensitivity and accuracy in qualitative and quantitative detection. However, complicated sample pretreatment and time-consuming analysis process restrict their applications in rapid and cost-effective testing. In contrast, SERS offers significant potential for addressing these limitations. To evaluate the performance of the nanoporous silver sheet, thiram was selected as the model molecule due to its widespread use as a protective fungicide in field crops, vegetables, and fruits, as well as the hepatotoxic risks associated with its metabolic degradation byproduct, such as carbon disulfide. Thus, the detection of thiram residues is essential, as they may pose significant health and environmental hazards, particularly concerning food safety and environmental monitoring.

**Fig. 2:**
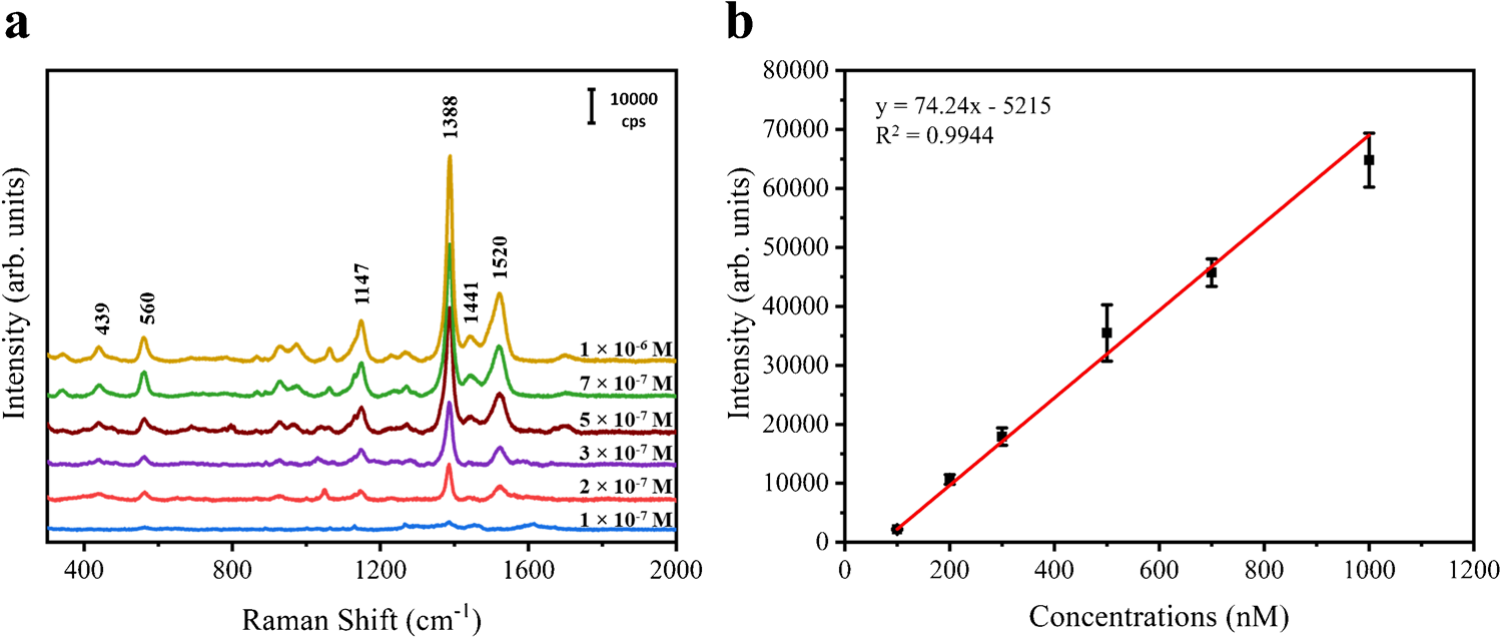
**a** SERS spectra of thiram solution at different concentrations acquired on the nanoporous silver sheet. **b** Quantitative analysis of the linear relationship between the concentrations of thiram solution and the intensities of the characteristic peak at 1388 cm^−1^

As shown in Fig. 2a, SERS spectra of thiram solution at different concentrations (from 100 to 1000 nM) were acquired. The prominent peak at 1388 cm^−1^, assigned to the C-N stretching and CH_3_ deformation, was chosen as a reference peak of thiram which was still detectable with a concentration down to 100 nM (0.024 ppm). Based on the peak at 1388 cm^−1^, it is found that the Raman intensities and the corresponding concentrations showed a linear relationship within the measured scale. This evidence indicates this nanoporous silver sheet is appropriate for quantitative analysis and its structure is generally uniform.

**Fig. 3:**
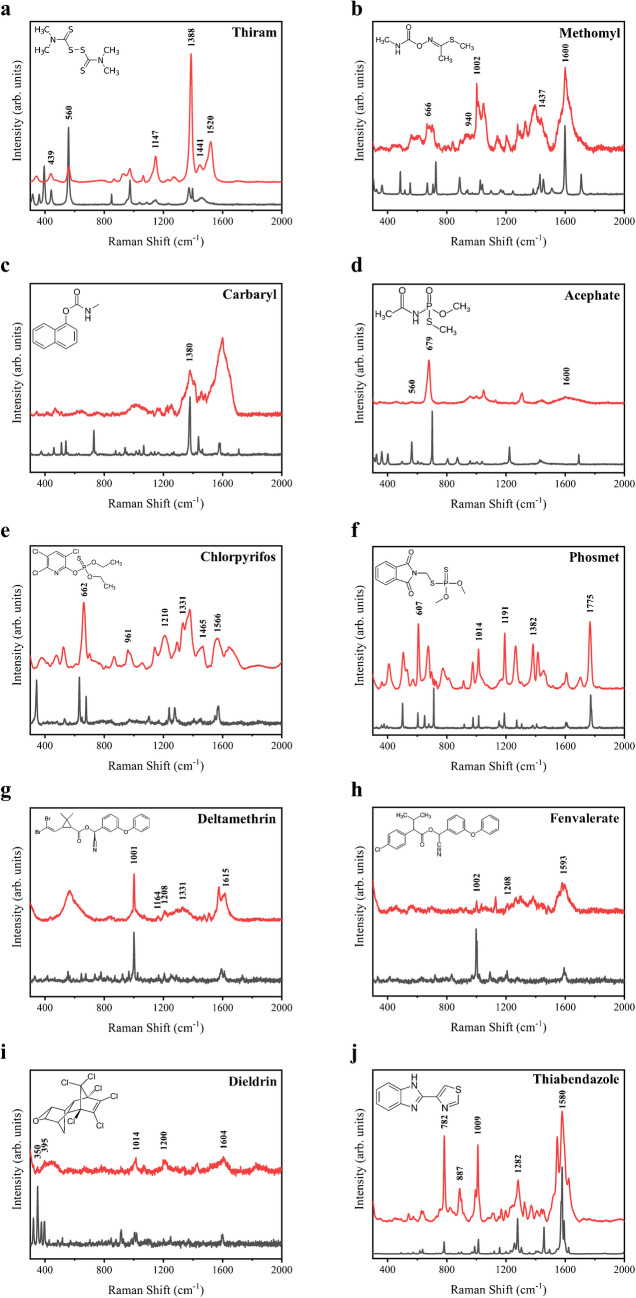
Normal Raman spectra (black curves) of solid samples and SERS spectra (red curves) of 10 µM solution of 10 different kinds of pesticides, including **a** thiram, **b** methomyl, **c** carbaryl, **d** acephate, **e** chlorpyrifos, **f** phosmet, **g** deltamethrin, **h** fenvalerate, **i** dieldrin, and **j** thiabendazole

According to the chemical compositions and active groups of these pesticides, they can be categorized into several groups: carbamates, organophosphorus, pyrethroids, organochlorines, and benzimidazoles. Carbamate insecticides kill insects by inactivating the delivery of the nerve signals and cause brain damage upon high dose exposure [21]. It is noted that GC–MS cannot detect thiram directly [22], while our nanoporous silver sheet has been demonstrated to directly detect the thiram, exhibiting characteristic peaks similar to those found in its normal Raman spectrum (Fig. 3a). Moreover, it is also applicable to other carbamate pesticides, for example, methomyl and carbaryl. As shown in Fig. 3b, the characteristic peaks of methomyl at 666, 940, 1002, 1437, and 1600 cm^−1^ attributed to aliphatic C-S stretching, C-H out-of-plane bending, ring breathing, asymmetric CH_3_ bending, and C = N stretching, respectively [23, 24]. The SERS spectrum of carbaryl (Fig. 3c) shows the distinct band at 1380 cm^−1^ assigned to the symmetric vibration of the naphthalene ring [25, 26], which is often chosen to identify the carbaryl residues [27]. Organophosphorus pesticides have been widely used in agricultural production. The common feature of the organophosphorus pesticides is the functional thiophosphoryl (P = S) groups, which can threaten human health by acute poisoning [28]. The nanoporous can detect organophosphorus pesticides, for example, acephate, chlorpyrifos, and phosmet, mainly due to the affinity of the phosphoric ester group or the thiophosphate group to substrate. As shown in Fig. 3d, the characteristic SERS peaks of acephate were at 560, 679, and 1600 cm^−1^, which were assigned to the P-S-C bending, P-O-C stretching, and ketone mode, respectively [25, 29]. Chlorpyrifos is one of the best-selling pesticides [30], but when its residues are accumulated to a certain amount, it may cause health problem related to neurodevelopment [31]. The SERS characteristic peaks of chlorpyrifos at 662, 961, 1210, 1331, 1465, and 1566 cm^−1^ (Fig. 3e) assigned to P = S, Cl-ring wagging, C-H bending, C = C asymmetric stretching, C = C stretching, and ring stretching can be detected on the nanoporous. Phosmet is another popular organophosphorus pesticide. The intense peaks of phosmet (Fig. 3f) at 607, 1014, 1191, 1382, and 1775 cm^−1^ came from C = O in-plane deformation, P-O-C asymmetric stretching, P-O-CH_3_ out-of-plane deformation, CH_3_ in-plane deformation, and C = O stretching [32, 33]. Pyrethroid pesticide is frequently utilized as insecticide which exerts strong effect of contact killing and stomach poisoning in controlling pests. Exceeded residues of pyrethroid is believed to be toxic to the reproductive system and disruptive to endocrine function [34]. Deltamethrin is one of the most toxic pyrethroids, which could cause upper respiratory irritation, dizziness, and vomiting after accumulation in the human body [35]. As shown in Fig. 3g, the characteristic SERS peaks of deltamethrin can be detected at 1001, 1164, 1208, 1331, and 1615 cm^−1^, which were assigned to benzene ring stretching, C-H deformation, C–C stretching, C-H deformation, and C = C stretching [36]. Fenvalerate is another popular pyrethroid pesticide used in agriculture, but few studies have investigated it by SERS. The nanoporous can detect the main characteristic peaks of fenvalerate at 1002, 1208, and 1593 cm^−1^, which is consistent with its normal Raman spectrum (Fig. 3h). Organochlorine is a general pesticide to kill target insects via disrupting their nervous system [37]. Due to its strong stability and acute toxicity, the organochlorine pesticide is hard to degrade in the environment and can be exposed to humans through the food chain bioaccumulation, which could stimulate the central nervous system and cause the disorder of epencephalon and even cancer [38, 39]. Dieldrin is a common organochlorine which is widely used as an insecticide and pesticide. Recently, the exposure of dieldrin has been correlated to the incidence of Parkinson’s disease [40]. The SERS spectrum of dieldrin (Fig. 3i) shows the distinct band at 350 and 395 cm^−1^ assigned to C–Cl stretching and deformation, which is often used to identify the organochlorine residues. Other SERS peaks of dieldrin are located at 1014, 1200, and 1604 cm^−1^, which are assigned to C–C stretching, C-H rocking, and C = C stretching [41]. Benzimidazole is a class of fungicides that can control fungal pathogens and has been effectively used in food crops by nuclear division blockage and DNA synthesis inhibition [42]. Thiabendazole is one of the benzimidazoles which is applied to control fungal diseases in fruits and vegetables. As shown in Fig. 3j, main characteristic SERS peaks for thiabendazole at 782, 887, 1009, 1282, and 1580 cm^−1^ are determined, which are assigned to C-S stretching, C–C-S bending, C-N stretching, ring stretching, and C = N stretching [43]. Therefore, our nanoporous silver sheet is widely applicable to different types of pesticides, including carbamates, organophosphorus, pyrethroids, organochlorines, and benzimidazoles, which can act as an effective SERS platform for hazardous pesticide detection.

**Fig. 4:**
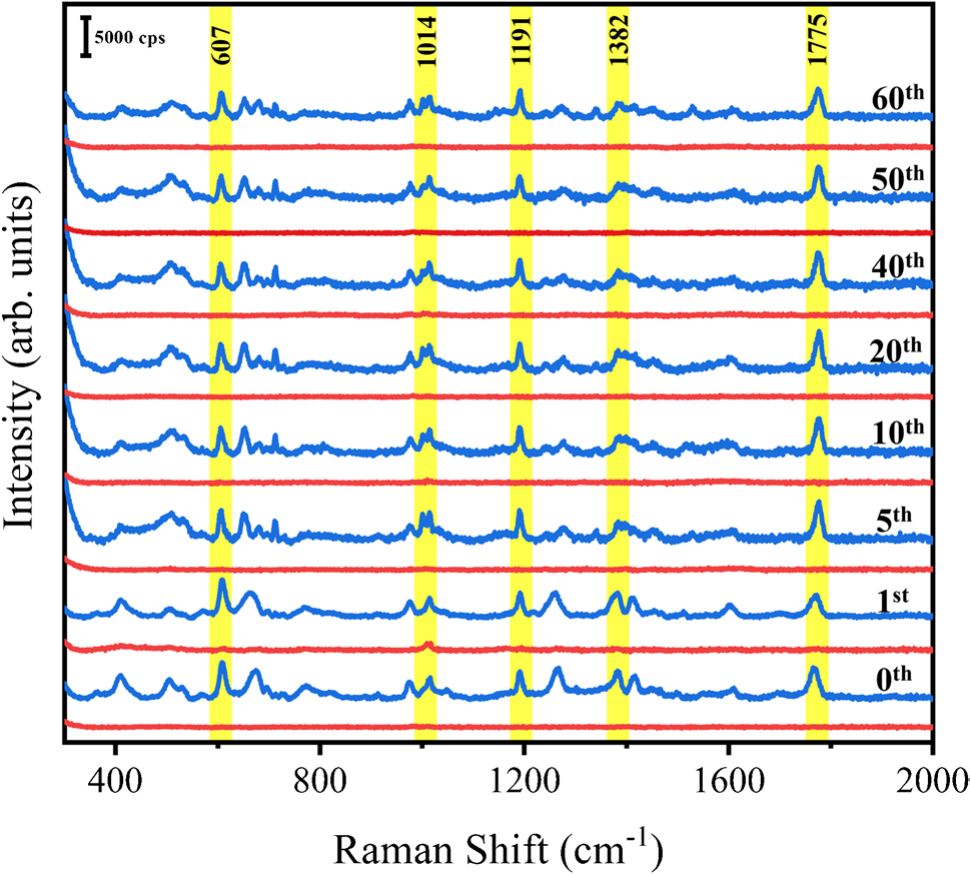
Reusability test of the nanoporous silver sheet for SERS detection of 10 µM phosmet solution (blue) and background spectra after ethanol wash (red) in 60 cycles

Considering the importance of cost-effective detection in practical applications, we evaluated the reusability of the nanoporous sheet. To begin with, a fresh nanoporous silver sheet was immersed in the analyte solution (10^–5^ M phosmet) for 5 min to facilitate the adsorption of phosmet into the nanopores. Afterwards, the sheet was removed and dried in room temperature for SERS measurement. The main characteristic peaks of phosmet at 607, 1014, 1191, 1382, and 1775 cm^−1^ are clearly observed (Fig. 4, the bottom blue spectrum as the starting cycle). After that, a one-step cleaning process was performed by simply immersing the used nanoporous silver sheet in ethanol for 4 h under continuous stirring to clean the adsorbed phosmet. The cleaned nanoporous silver sheet was then dried for background check by SERS. A spectrum with no detectable signal of phosmet was observed (Fig. 4, the bottom red spectrum as the first cycle of cleaning), confirming that the attached phosmet was removed thoroughly after the cleaning procedure. In 60 successive cycles, phosmet was well detected (Fig. 4, blue spectra). More importantly, the intensities of phosmet and the sheet in each cycle were well maintained (Figs. S2 and S3), which can be attributed to the robust structure of the nanoporous sheet. Notably, the efficiency of washing an analyte off the porous silver substrate is indeed influenced by its adsorption coefficient on silver. Phosmet was selected as an example because its structure includes a phenyl ring, a hydroxyl group, a phosphorus atom, and two sulfur atoms, which can form coordinate covalent bonds with silver to enhance adsorption. The effective removal of phosmet suggests that other pesticides, generally with weaker adsorption affinities, would be more readily washed off from the substrates using the same rinsing method. Additionally, no significant changes in the nanoporous structures were observed in a 72-h rinsing test (Fig. S4). These results demonstrate the consistent SERS sensitivity and excellent stability of the nanoporous silver sheet in reuses, providing a convenient and cost-effective solution for SERS applications.

**Fig. 5:**
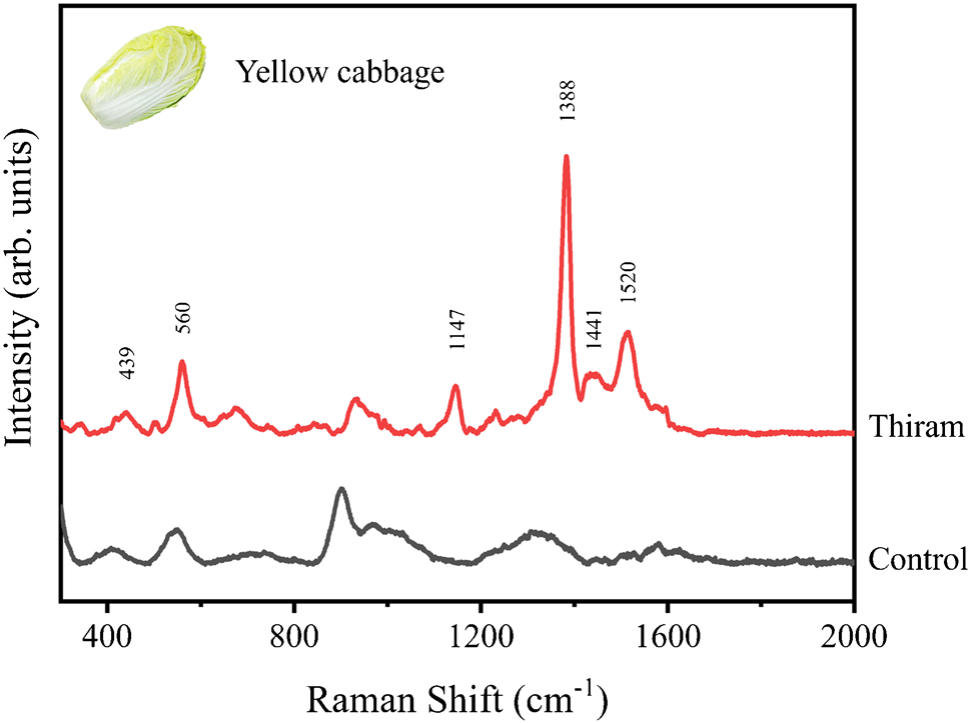
SERS detection of thiram residues on leaves of a yellow cabbage

To assess the availability and practicality of our nanoporous silver sheet for practical applications, we further conducted a case study in detecting the pesticide residue on the leaves of yellow cabbage. During the growth, storage, and shipment of yellow cabbage, thiram is a widely used pesticide to control fungal diseases. However, the extensive use of thiram raises concerns about human health, yet it cannot be directly detected by GC–MS [22]. This highlights the necessity to explore the potential of SERS for pesticide determination in practical implementation. To simulate the application of thiram during the cultivation of yellow cabbage, we first added 100 µL of 1 µM thiram solution onto a 1 cm^2^ area of a yellow cabbage leaf (see the inset in Fig. 5). Then the thiram-sprayed leaf was dried under ambient conditions. To extract the pesticide residue from yellow cabbage for SERS detection, 20 μL ethanol was dispersed on the surface of the thiram-sprayed leaf. Notably, depending on the type of vegetables and pesticides, a higher volume of ethanol would facilitate more comprehensive extraction and detection. After about 2 min, the solution on the leaf was collected into a small tube and the nanoporous silver sheet was totally immersed in the solution. The nanoporous silver sheet was then dried at room temperature for SERS measurement. As shown in Fig. 5, the SERS spectrum of the extract solution shows the prominent characteristic peaks (439, 560, 1147, 1388, 1441, and 1520 cm^−1^) of thiram, which is in good agreement with the SERS spectrum of thiram in Figs. 2 and 3a. Control experiment with the adding of water was also carried out and the corresponding spectrum was presented (Fig. 5, black) for comparison. Note that the applied concentration of thiram (1 µM) is much lower than the maximal residue limit of 7 ppm (29 µM) prescribed by the US Environmental Protection Agency (EPA) [44, 45]. Referred to the quantitative analysis in Fig. 2, it is believed that a lower concentration of thiram should still be detectable. This case study indicates that our nanoporous silver sheet shows a high potential in practical applications.

## Conclusions

In summary, we utilized a straightforward chemical redox method to fabricate the novel nanoporous silver sheet for a highly sensitive, background-free, and reusable SERS platform. The nanoporous silver sheet features a multilayered 3D structure of uniform coral rock–like nano-silver rods, which provides numerous SERS-active hotspots to achieve a detection limit of 1 × 10⁻⁷ M (24 ppb) for thiram. Furthermore, it exhibits universal detection capability across a wide range of pesticide compounds, including carbamates, organophosphorus, pyrethroids, organochlorines, and benzimidazoles, with its practical viability confirmed through testing on real-world samples like yellow cabbages. More importantly, owing to its robust structure and intricate uniformity, this SERS platform demonstrates high reusability, maintaining consistently outstanding performance for up to 60 cycles of continuous SERS measurements with simple rinsing procedures. Leveraging its easy fabrication, excellent sensitivity, and high reusability, the nanoporous silver sheet presents significant potential as a promising SERS substrate for cost-effective trace analysis, which will promote adoption and practical application of SERS-based analytical techniques in various fields.

## Supplementary Information

Below is the link to the electronic supplementary material.

Supplementary file1 (DOCX 1918 KB)

## Data Availability

All the original data for this article are available at DataSpace@HKUST at 10.14711/dataset/BP30DS.
