# Supplementary material for: Simple strategy for preparing nanoporous silver sheets as reusable SERS substrates for trace analysis with up to 60 reuses
Source: Anal Bioanal Chem. 2025 May 22;417(16):3611–9. doi: 10.1007/s00216-025-05903-2 (PMC12206212; doi:10.1007/s00216-025-05903-2)
Supplement: Supplementary file 1 — Supplementary file1 (DOCX 1918 KB) [file 216_2025_5903_MOESM1_ESM.docx]

**Supplementary Information**

**Simple strategy for preparing nanoporous silver sheets as reusable SERS substrates for trace analysis with up to 60 reuses**

Hongni Zhu^1,2^, Vince St. Dollente Mesias^1^, Xin Dai^1^, Wenting Qiu^1^, Xiaobin Yao^1,*^, Jinqing Huang^1,*^

^1^Department of Chemistry, The Hong Kong University of Science and Technology, Clear Water Bay, Hong Kong, China

^2^Research Center for Biomedical Optics and Molecular Imaging, Key Laboratory of Biomedical Imaging Science and System, Shenzhen Institutes of Advanced Technology, Chinese Academy of Sciences, Shenzhen 518055, China

*Corresponding authors:

Xiaobin Yao

[xiaobinyao@ust.hk](mailto:xiaobinyao@ust.hk)

Jinqing Huang

[jqhuang@ust.hk](mailto:jqhuang@ust.hk)


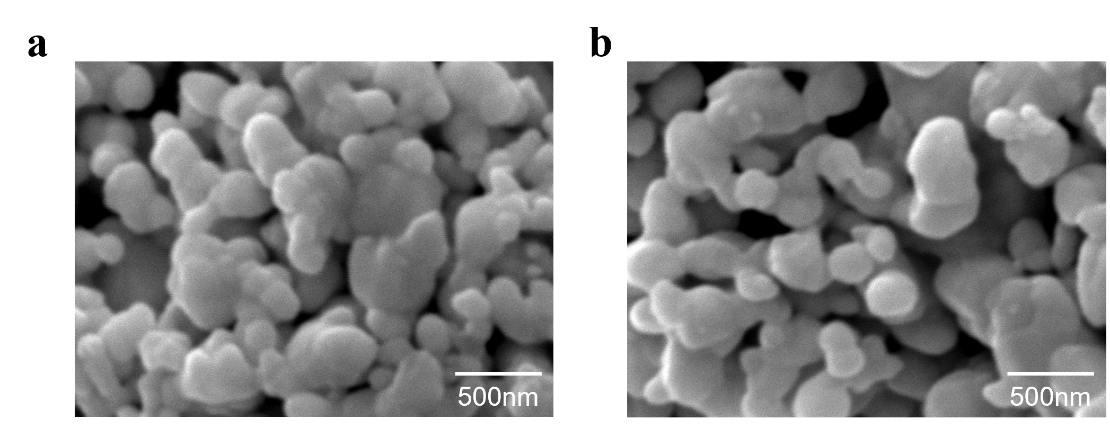


**Fig. S1** SEM images of a nanoporous silver sheet from different locations.


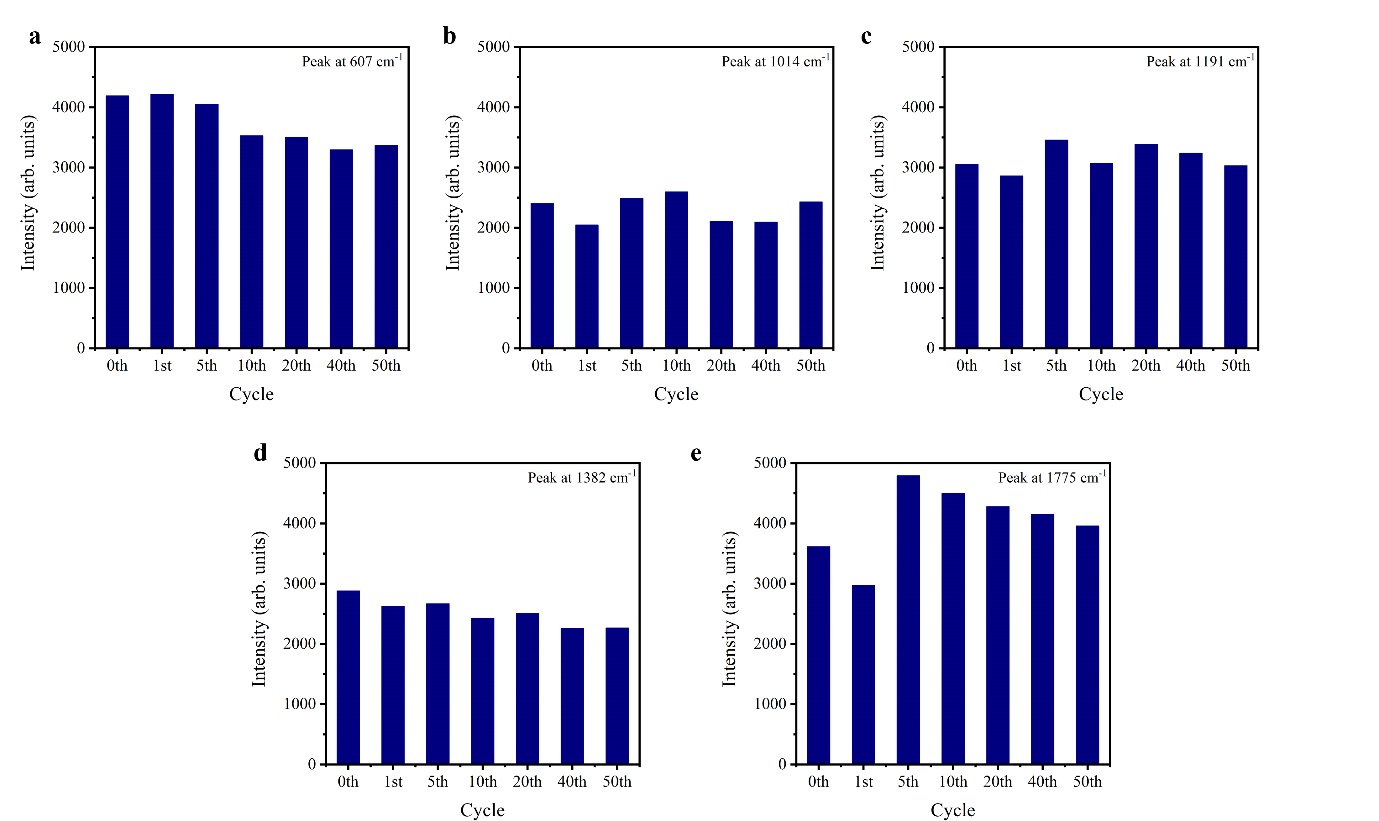


**Fig. S2** Intensity histograms of selected characteristic peaks of phosmet on a nanoporous silver sheet in 60 cycles.


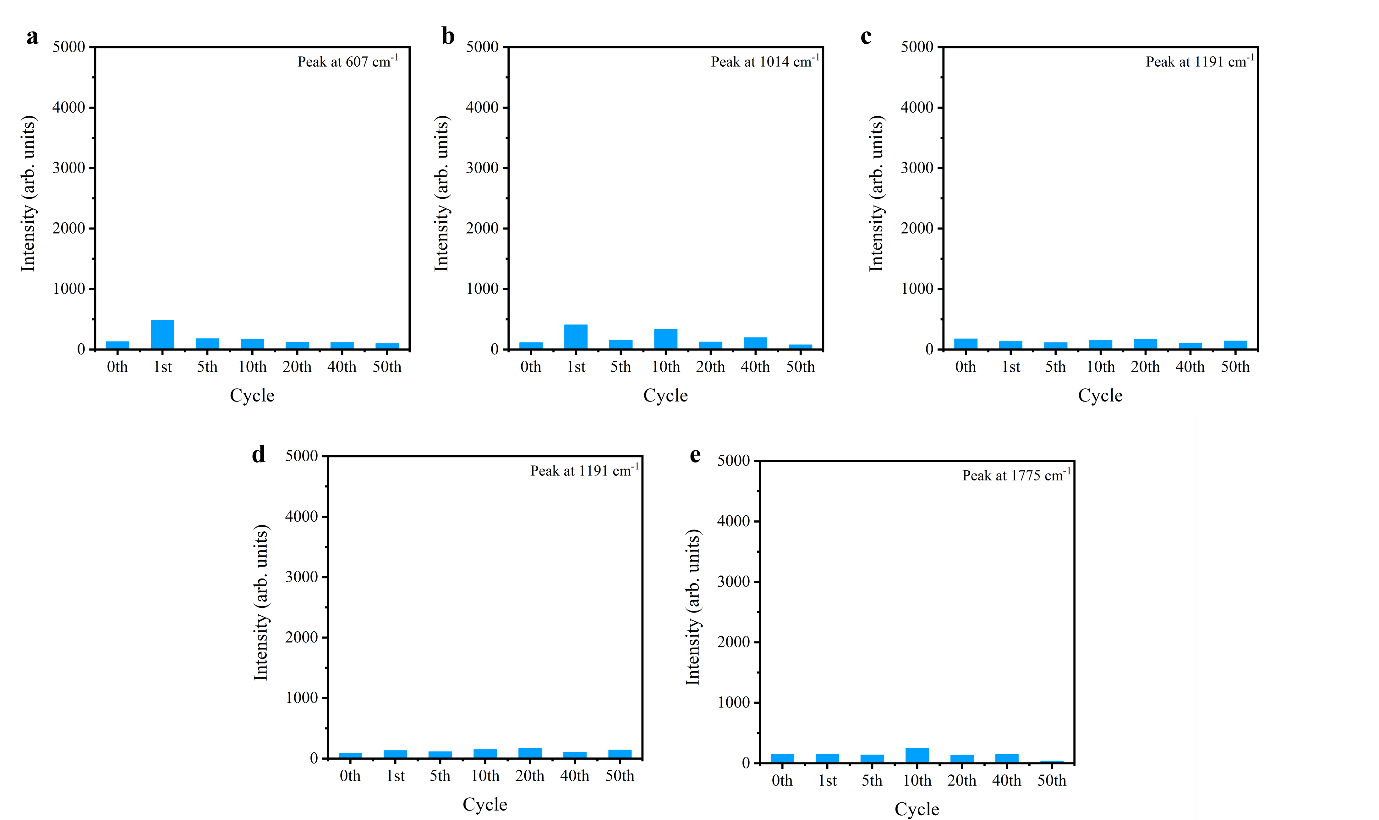


**Fig. S3** Intensity histograms of the nanoporous silver sheet at corresponding peaks in 60 cycles.


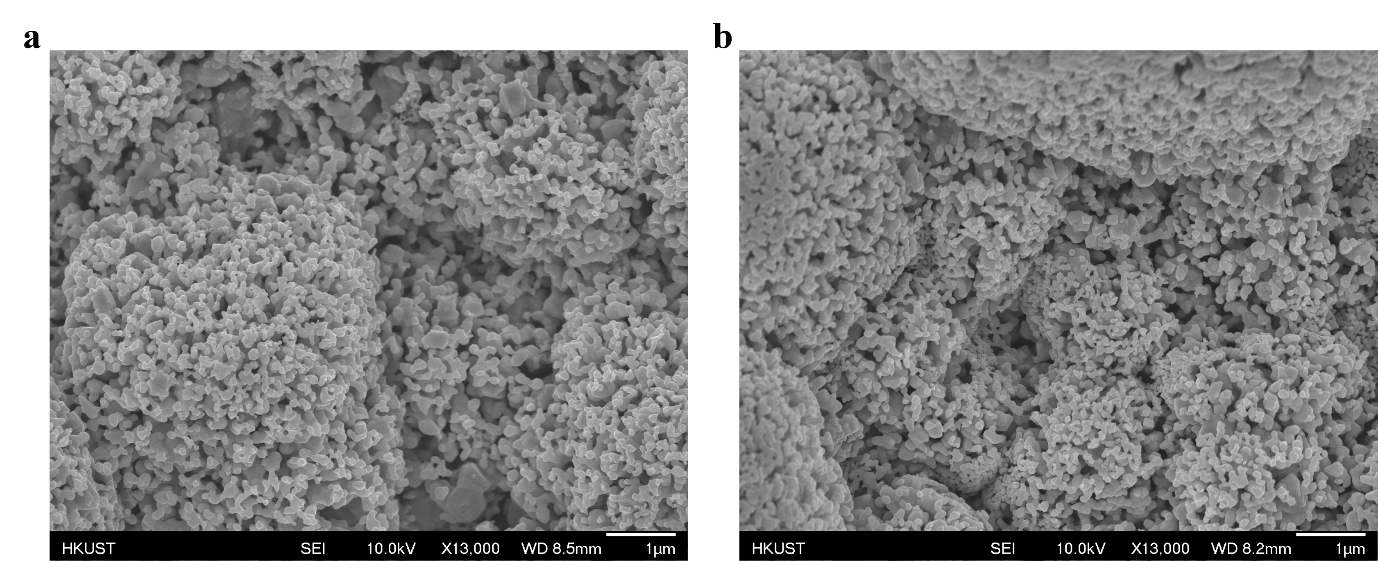


**Fig. S4** SEM images of a nanoporous silver sheet before (a) and after 72 hours rinsing (b) with ethanol. Images were acquired from different regions.
